# Hydrogen-Bond Acidic Materials in Acoustic Wave Sensors for Nerve Chemical Warfare Agents’ Detection

**DOI:** 10.3390/s24082477

**Published:** 2024-04-12

**Authors:** Michał Grabka, Krzysztof Jasek, Zygfryd Witkiewicz

**Affiliations:** Institute of Chemistry, Faculty of Advanced Technologies and Chemistry, Military University of Technology, 00-908 Warsaw, Poland; krzysztof.jasek@wat.edu.pl (K.J.); zygfryd.witkiewicz@wat.edu.pl (Z.W.)

**Keywords:** SAW, QCM, acoustic wave sensor, DMMP, nerve CWAs

## Abstract

The latest trends in the field of the on-site detection of chemical warfare agents (CWAs) involve increasing the availability of point detectors to enhance the operational awareness of commanders and soldiers. Among the intensively developed concepts aimed at meeting these requirements, wearable detectors, gas analyzers as equipment for micro- and mini-class unmanned aerial vehicles (UAVs), and distributed sensor networks can be mentioned. One of the analytical techniques well suited for use in this field is surface acoustic wave sensors, which can be utilized to construct lightweight, inexpensive, and undemanding gas analyzers for detecting CWAs. This review focuses on the intensively researched and developed variant of this technique, utilizing absorptive sensor layers dedicated for nerve CWAs’ detection. The paper describes the mechanism of the specific interaction occurring between the target analyte and the sensing layer, which serves as the foundation for their selective detection. The main section of this paper includes a chronological review of individual achievements in the field, largely based on the peer-reviewed scientific literature dating back to the mid-1980s to the present day. The final section presents conclusions regarding the prospects for the development of this analytical technique in the targeted application.

## 1. Introduction

Due to the ongoing threat of chemical warfare on the battlefield, the detection of chemical warfare agents (CWAs) remains a matter of critical importance. Currently, there are many point detectors based on well-established analytical techniques such as mass spectrometry [1,2], ion mobility spectrometry [3,4], or flame photometry [5], which serve this purpose. Despite the availability of various types of devices for such analyses, some applications still remain beyond the reach of currently available commercial equipment. Examples include wearable detectors integrated into clothing or protective gear [6,7,8], onboard analyzers deployed on unmanned aerial vehicles [9,10], or distributed sensor networks [11]. Consequently, to address this gap, research is being conducted worldwide to develop devices based on alternative techniques characterized by low unit costs and minimal operational requirements, while ensuring durability and desired metrological properties.

An analytical technique that meets these requirements well is the gas sensors with acoustic wave. The first application of sensors of this type for detecting organic vapors was described as early as 1964 [12]. In this work, quartz microbalances were used, onto which thin liquid films (including elastomers) were deposited. The sorption of molecules of compounds present in the gaseous environment of the sensor caused an increase in the mass of the film through absorption. This results in a decrease in the frequency of the vibrations of the system proportional to the concentration of molecules in the gas.

Over the years, various modifications of King’s original idea have emerged to ensure effective detection of CWAs, especially the most dangerous group, which is the nerve agents. The available literature provides numerous examples of sensors equipped with various types of adsorptive layers utilizing metal oxides [13,14,15], transition metal dichalcogenides (TMDs) [16], carbon nanomaterials [17,18,19], and other functional materials [20], including biomolecules [21]. Furthermore, numerous studies have demonstrated the application of conductive polymers and other macromolecules. These investigations exploit the significant sensitivity of acoustic wave transducers to detect changes in the electrical parameters of the sensor layer [22,23,24,25,26]. Despite the numerous solutions available, the most frequently researched and utilized group of materials for the sensor layers are non-conductive polymers and other elastomeric macromolecules that absorb analytes within their volume (similarly to the initial King sensors). Thanks to the appropriate design (chemical modifications), these materials selectively interact with organophosphate analyte molecules, effectively sorbing them.

This review focuses on the application of polymers and non-conductive macromolecules in acoustic wave sensors for detecting nerve CWAs and their simulants. This paper elucidates the mechanism of the specific interaction between organophosphorus compounds and the sensing layer, which forms the basis for their selective detection in gas. This description utilizes the linear solvation energy relationship (LSER) model, widely employed in the study of absorption materials (a description of the model is provided in Section 1.2).

The main part of this paper provides a comprehensive review of significant milestones in the field. These achievements are mainly sourced from peer-reviewed scientific journals and cover the period from the mid-1980s to the present day. The final section of this article discusses the development potential of this analytical technique for the detection of CWAs in real field conditions.

### 1.1. Sensors Equipped with Absorptive Layers

The absorptive sensor layers are made of non-volatile organic liquids or polymers that, at the sensor’s operating temperature, exist in a liquid (viscoelastic) state and absorb analyte within their volume. This process can be thought of as dissolution of the solute in the liquid, which can be accompanied by the adsorption of the analyte onto the surface of the gas–liquid or solid substrate–liquid interface. The distribution of a specific analyte between the bulk sensor layer and the gaseous environment of the sensor can be described by Henry’s law [27]:

K = C_S_/C_V_, (1)

where K—partition constant; C_S_—the concentration of the analyte in the sensor layer; C_V_—the concentration of the analyte in the ambient gas.

The response of a sensor (R) equipped with such a layer is proportional to the amount of absorbed vapors, which can be expressed as a function of the partition constant:

R = SC_V_K, (2)

where S—sensor sensitivity factor.

The absorption of the analyte in the sensor layer causes the following: additional mass loading on the surface of the transducer, a change in its stiffness resulting from polymer swelling (changes in the compressibility modulus and shear modulus), and a change in the electrical parameters of the layer (mainly electrical conductivity but also the charge carrier diffusion constant and electrical permeability of the layer). Considering the basic types of acoustic wave sensors, it can be said that in the case of sensors with bulk acoustic wave (quartz microbalances (QCMs) or Film Bulk Acoustic Resonator (FBAR), type structures [28]), the sensitivity factor S in Equation (2) takes into account the sensor’s sensitivity to changes in mass loading and changes in the stiffness of the layer. In the case of surface acoustic wave (SAW) sensors (devices with Rayleigh, Love, and Shear Horizontal SAW types), the sensitivity parameter additionally takes into account the sensitivity to changes in the electrical parameters of the layer. In cases where the SAW sensor layer exhibits low surface conductivity (below approximately 1 × 10^−8^ S [29], which is fulfilled in the majority of isotropic elastomers considered in this review), the sensor is not sensitive to changes in the electrical parameters of the layer. In such cases, the SAW sensor signal is generated through mass loading and changes in the viscoelastic parameters.

### 1.2. Linear Solvation Energy Relationship (LSER) Model

In the case of absorptive sensor layers, analyte sorption in the polymer is the result of intermolecular interactions occurring in the liquid solution. These interactions include hydrogen bonding formation and Van der Waals forces: induced dipole–induced dipole interactions (“London” forces), permanent dipole–permanent dipole interactions (“Keesom” forces), and permanent dipole–induced dipole interactions (“Debye” forces) [30]. The selectivity and maximization of the sorption capacity are achieved by adjusting the solvation properties of the polymer to a specific analyte (more often groups of analytes). In practice, this involves modifying polymer materials with specific functional groups introduced into the structure of functional substituents, which exhibit the ability to interact with the target analyte according to a specific mechanism. For example, cyanide groups increase the ability for polar interactions, aromatic compounds and heavy halides enhance the ability for polarized interactions, and alcohols and phenols increase the ability for interactions via hydrogen bonding, where the sorption material acts as a hydrogen atom donor, etc. [31].

One of the semi-empirical models allowing for the prediction of the partition coefficient values and widely used in the literature of acoustic wave sensors equipped with absorptive layers is the LSER [30]. The LSER model quantitatively characterizes the solvation properties of the dissolved substance (analyte) and the solvent (polymer) using a set of coefficients. Each coefficient can be related to a specific type of intermolecular interactions occurring in the solution and influencing solubility. Expressing the value of the analyte partition coefficient in the liquid–gas system according to the LSER model can be written as follows [32]:

logK = c + eE + sS + aA + bB + lL,(3)

In Equation (3), the coefficients denoted by lowercase letters correspond to the solvent. Their individual meanings are as follows: e—ability to engage in polarized interactions, s—ability to engage in polar interactions, a and b—ability to act as a hydrogen acceptor and donor during hydrogen bonding formation, and l—ability to engage in dispersion interactions. c is a system constant resulting from the mathematical method of determining the remaining coefficients. Descriptors of the solute, denoted by capital letters, correspond to the complementary characteristics of the dissolved substance. According to Equation (2), the value of the partition coefficient (which is proportional to the absorbed substance amount) is the sum of contributions from individual intermolecular interactions between the polymer and the analyte in the solution. The contribution of each type of interaction, in turn, is the product of the complementary LSER coefficient of the polymer and the analyte descriptor.

### 1.3. Mechanism of Sorption of Organophosphorus Compounds

A common characteristic of nerve CWAs is their high ability to form hydrogen bonds, where they act as hydrogen acceptors (base of hydrogen bonding). At the same time, these compounds do not have the ability to act as a hydrogen atom donor in hydrogen bonding [33]. This thesis is confirmed by the values of LSER model descriptors for individual compounds belonging to this group. The descriptors’ values determined by quantitative structure property relationship (QSPR) calculations (empirical values are not available in the literature) are presented in Table 1. Additionally, the table includes LSER descriptors for dimethyl methylphosphonate (DMMP), frequently used in studies as a simulant for live CWAs (a substance structurally similar but significantly less toxic).

**Figure 1:**
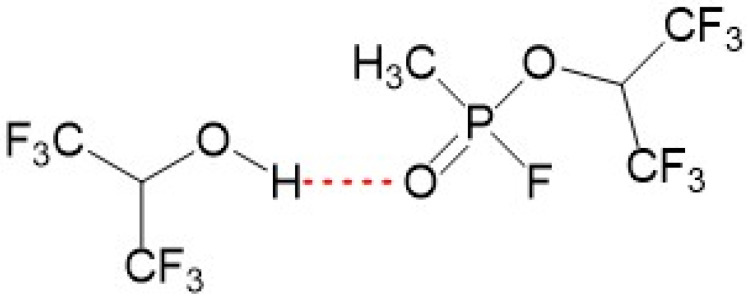
Formation of hydrogen bond between hexafluoroisopropanol (hydrogen-bonding acid—HBA) and sarin (hydrogen-bonding base—HBB).

In Table 1, it is observed that all substances listed have high values of descriptor B, representing basicity in terms of hydrogen bonding. At the same time, the values of A (representing acidity) are equal to 0, except for GA tabun for which A = 0.05, due to the presence of an amine group. As a result of such solvation interactions, organophosphorus compounds are considered relatively specific, and the formation of basic hydrogen bonds by them is acknowledged as a selective mechanism for the sorption of these compounds. Figure 1 illustrates the formation of a hydrogen bond between GB (sarin, a hydrogen-bonding base) and the hydrogen-bonding acid.

For this reason, materials with a high capacity to form acidic hydrogen bonds are used for the selective sorption of organophosphorus compounds. These properties are exhibited in molecules containing a hydrogen atom covalently bonded to an element with high electronegativity, such as oxygen or nitrogen. Examples of such substances include compounds containing a hydroxyl group. Within this group, the hydrogen atom is covalently bonded to an oxygen atom. Owing to the considerable electronegativity of the oxygen atom, it carries a partial negative charge, while the hydrogen atom bears a partial positive charge.

However, not all chemical compounds containing a hydroxyl group are equally strong acids (and weak bases) in terms of hydrogen bonding. What determines effective acidity is, among other factors, the magnitude of the partial positive charge accumulated on the hydrogen atom and the negative charge on the oxygen atom of the hydroxyl group. According to the literature data [33,36], the effective acidity of a molecule can be increased by introducing fluorine atoms into its structure. Fluorine, as the most electronegative element (EN = 3.98 [37]), induces the electron-withdrawing effect from the rest of the molecule, including the hydroxyl group, influencing the distribution of the partial charge. Consequently, the acidity of the hydrogen atom in the hydroxyl group increases (increase in partial positive charge) while the basicity of the oxygen atom decreases. Due to their solvating properties, functional substituents containing fluoroalcohol and fluorophenol groups are used to obtain acidic polymers for sensor applications.

It is also worth mentioning that in the detection of nerve CWAs using acoustic wave sensors, materials characterized by dominant types of interactions other than the formation of acidic hydrogen bonds were also used. This approach was utilized in sensor matrices, employing materials spanning the full spectrum of intermolecular interactions [38,39,40]. The purpose of this is to diversify sorption properties and increase selectivity. Notable examples of such materials are polymers with high overall polarity (indicated by a high value of the s coefficient). These materials possess a strong ability to adsorb nerve CWAs through polar interactions, which are less selective than the formation of acidic hydrogen bonds [41].

An essential aspect of polymer materials concerning their application as absorption layers in gas sensors is their glass transition temperature (T_g_). Below this temperature, the sensor layer remains in a glassy state, where the diffusion coefficient of analyte molecules is significantly slower compared to temperatures above T_g_. A low diffusion coefficient prolongs the time required to reach sorption equilibrium, thereby affecting the sensor’s response dynamics. Once T_g_ is surpassed, the diffusion coefficient rapidly increases, leading to a fast sensor response to changes in analyte concentration.

Simultaneously, raising the temperature of the sensing layer (the operational temperature of the sensor) above T_g_ causes a decrease in the analyte partition constant (K) between the polymer and the gas phase. This decrease is attributed to the weakening of intermolecular interactions between analyte molecules and the polymer. The temperature-dependent change in K can be described by an Arrhenius-type relationship [42]:

K = K_0_ × e^−(ΔHC+ΔHM)/RT^ (4)

where the K_0_ term is independent of temperature, ΔH_C_ is the molar heat of condensation, ΔH_M_ is the partial molar heat of mixing, and R and T are the gas constant and the absolute temperature, respectively. The consequence of reducing the polymer’s ability to absorb vapors is a decrease in the sensitivity of the sensor. Taking the above into account, it is desirable that the designed polymer materials have a T_g_ value as low as possible in order to achieve good sensitivity and response dynamics.

## 2. Review of Hydrogen-Bond Acidic (HBA) Polymers

Polymers exhibiting an enhanced capacity to form acidic hydrogen bonds are commonly referred to as hydrogen-bond acidic (HBA) polymers in the literature. Structurally, they share functional groups, typically fluoroalcohol and fluorophenol groups, facilitating the formation of hydrogen bonds where the polymer serves as a proton donor. Over time, these materials have evolved based on polymers featuring carbon, polysiloxane, or carbosilane backbones. Therefore, categorizing these materials based on the polymer chain type is most practical. Following this criterion, HBA polymers are categorized into organic polymers (with carbon chains) and polymers containing silicon-based chains (polysiloxanes and carbosilanes).

A comprehensive review of such materials was published in 2008 [33]. This review encompasses advancements in HBA polymers from the initial reports in the mid-1980s to early 2007. However, there is a noticeable gap in literature reviews covering the period from 2007 to the present day. Hence, our review predominantly focuses on works published after 2007, with a brief overview of the most significant earlier publications.

### 2.1. Linear Organic Polymers

Chronologically, the first category of organic chain acidic polymers comprised derivatives of polystyrene modified with substituents containing a hexafluoroisopropanol-HFIP group (also known as hexafluorodimethyl carbinol group). The initial reports regarding the use of these materials for selectively sorbing organophosphorus compounds (DMMP) and their application in QCM sensors date back to 1984 [33,43], although the material itself was developed as early as 1980 [44]. Subsequent works on this subject were published in 1987 [45,46]. These studies described the investigations into the sorption properties of styrene copolymers and fluorocarbinol-substituted styrene. The research involved the use of inverse gas chromatography, calorimetry, and the measurement of DMMP vapor sorption on polymer layers deposited on quartz microbalances. The QCM measurements were conducted at a relatively high temperature (135 °C), where hydrogen bonding interactions are considerably weaker compared to lower temperatures.

**Figure 2:**
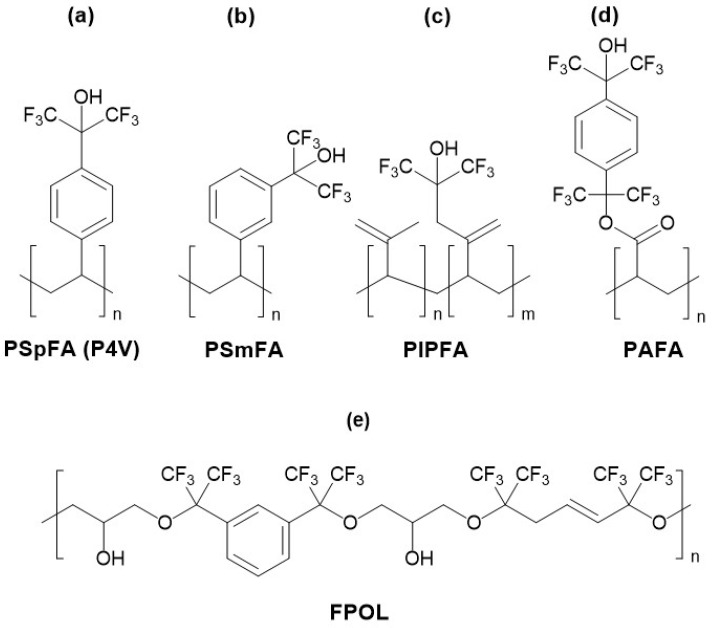
Structures of repeating units of some acidic organic polymers and acronyms used in the research papers: (**a**,**b**) hexafluoroisopropanol-HFIP modified polystyrenes (PSpFA, PSmFA), (**c**) HFIP modified polyisoprene (PIPFA), (**d**) HFIP modified polyacrylic (PAFA), and (**e**) fluoroepoxy resin (FPOL).

The expansion of research into acidic polymers utilizing polystyrene chains culminated in a study published by Snow et al. in 1991 [47]. This work outlined the synthesis and systematic investigation of an entire family of materials based on polystyrene (acronyms PSpFA, PSmFA, PSoFA), alongside polyisoprene (PIPFA) and polyacrylic (PAFA), all modified with the strongly acidic HFIP group. The structures of some of these materials are depicted in Figure 2a–d. The aforementioned study reported the results of DMMP detection using SAW sensors coated with the specified polymers. To determine the true impact of fluoroalcohol groups on DMMP sorption via hydrogen bond formation, additional measurements were conducted with materials where hydroxyl groups were deactivated through acetylation. The experiment confirmed the role of hydroxyl groups in the detection mechanism, as sensor signals with deactivated coatings were 5 to 10 times lower than those with free hydroxyl groups. Furthermore, infrared analysis was carried out to monitor changes in absorption bands arising from polymer hydroxyl groups during DMMP sorption. In the infrared spectra, clear shifts in bands characteristic of hydrogen bond formation were observed. Studies involving SAW sensors and infrared—IR—spectrum analysis demonstrated the pivotal influence of acidic hydroxyl groups in the sorption of basic compound vapors. The results of these investigations highlighted the effectiveness of modifying polymers with substituents possessing a high capacity for forming acidic hydrogen bonds in the fabrication of selective sorption materials. However, the polymer materials presented in the study exhibited relatively high glass transition temperatures (significantly above room temperature). In the case of modified polystyrene PSpFA and PSmFA, the glass transition temperatures were 122 and 84 °C, respectively; for modified polyisoprene PIPFA, the range was from 32 to 60 °C; and for the derivative of polyacrylic acid PAFA, it was 175 °C. As a result, their practical application in gas sensors was severely constrained by poor response dynamics.

Several years after the initial reports on acidic polystyrenes from 1984 [33,43], an acidic sorption material with an epoxy resin structure, designated by the acronym FPOL, was presented. The first reports of using this material for the detection of organophosphorus compounds using SAW sensors date back to 1986 [48]. The material itself had been developed much earlier (it was described by Field in 1976 [49]), but initially, its inventors did not intend it for sensor applications. The FPOL material fabricated in the early stages of research was actually a rather heterogeneous fluoroepoxy copolymer mixture of various isomers. The structure of the dominant repeat unit of FPOL is depicted in Figure 2e. In the FPOL structure, hydroxyl groups distributed along the carbon chain can be identified as active sites for forming acidic hydrogen bonds. Additionally, the polymer’s acidity is enhanced by the presence of fluorine and oxygen atoms inducing an electron-withdrawing effect. Subsequent research on FPOL led to the development of more homogeneous materials characterized by precisely controlled isomeric composition [50,51,52]. Nonetheless, these materials did not significantly differ in sorption properties or other physical properties from the original FPOL. It is worth noting that the FPOL materials family is characterized by acceptably low T_g_ values ranging from 10 to 45 °C.

The FPOL material belongs to one of the most intensively studied HBA polymers. Research related to FPOL has been conducted by scientific teams from the USA [47,48,53,54], France [50,51], and India [52]. To this day, this material is used as a reference in research on new acidic polymers.

Among the most significant research findings regarding FPOL-based acoustic wave sensors include those where FPOL was used in sensor arrays [55] as well as in individual sensors [51]. Grate et al. [55] reported an array of SAW sensors integrated into a complete gas analyzer equipped with an automated sampling system, including a preconcentrator and utilizing advanced methods of signal processing. The array described in this study consisted of four actively temperature-controlled acoustic delay lines with a resonant frequency of 158 MHz. SAW devices were coated with various sensor polymers such as poly(epichlorohydrin) (PECH), poly(ethylenimine) (PEI), ethyl cellulose (ECEL), and the acidic polymer FPOL (applied by the spray coating method, resulting in a resonance frequency decrease of approximately 250 kHz). The analyzer was capable of detecting both simulants (DMMP) and live CWAs (GB, VX, and sulfur mustard—HD) in the presence of various interferents (fuel vapors, exhaust fumes, smoke) and at different gas humidities. As expected, the FPOL sensor exhibited the highest sensitivity to organophosphorus compounds among the array elements. The FPOL sensor detected DMMP vapor signals (without preconcentration) at approximately 200 Hz for 0.12 mg/m^3^ and at 1300 Hz for 1.1 mg/m^3^ (however, it was assumed that the detection limit for DMMP was 1 mg/m^3^). The sensor also showed good response dynamics (signal stabilization occurred after 40–50 s; measurements were conducted at 30 °C). The measurements conducted in gases with varying humidity (from 0 to 80%) did not reveal a significant influence on the detection of organophosphorus compounds. However, the FPOL sensor exhibited strongly nonlinear concentration characteristics with the steepest slope for the lowest DMMP concentrations. In the case of detecting live CWAs, the FPOL sensor demonstrated even better properties than in the case of DMMP. Without preconcentration, the sensor allowed for the detection of GD and VX at concentrations of 0.5 mg/m^3^ or lower regardless of gas humidity.

The analyzer utilized a thermally desorbed preconcentrator with a solid Tenax GC bed. This component was integrated into the automated sampling system and enabled operation in 2 and 14 min cycles. The use of the preconcentrator significantly lowered the detection limits for organophosphorus compounds and reduced the impact of interferents. The study achieved approximately a 10-fold increase (2100 Hz for 0.12 mg/m^3^) in the DMMP signal for the 2 min cycle and about a 90-fold increase (18,000 Hz for 0.12 mg/m^3^) for the 14 min cycle (concentration enhancement factors for initial, linear range were higher, approximately 20 and 200). The use of preconcentration enabled the detection of organophosphorus compounds at concentrations around 0.01 mg/m^3^ (for GB and VX with a 2 min preconcentration cycle), significantly lower than the 10 min AEGL-2 levels [56] often assumed as the detectability requirement for military devices for detecting chemical warfare agents. The FPOL sensor signal remains stable for at least 7 months.

In 2000, Rebière et al. [51] reported the results of testing FPOL sensors for sarin vapors and presented a systematic analysis regarding the influence of gas humidity and measurement temperature on the sensor performance. In this study, SAW sensors with acoustic delay lines operating at a frequency of 98 MHz were used, onto which FPOL layers were deposited using the spray technique. The applied layers varied in thickness and caused a decrease in the center frequency by approximately 50 to 220 kHz. Measurements were conducted at temperatures of 30, 35, and 40 °C. The research findings revealed that the sensor response time at 30 °C was very long and entirely unacceptable for detecting such hazardous substances (depending on the concentration, a steady-state signal was reached after 40–55 min). This prolonged response time was due to the fact that the polymer was in a glassy state at this temperature (the T_g_ for the tested FPOL was estimated at 30–35 °C). Upon raising the temperature to 35 and 40 °C, the sensor response time was drastically reduced (steady state reached after approximately 4–6 min). Simultaneously, the increase in temperature (especially to 40 °C) resulted in a significant decrease in sensitivity due to the analyte’s solubility decrease in FPOL. The analysis of gas humidity’s impact on sensor signals showed that in air with a relative humidity of 60%, there was a substantial increase (by 50%) in sensitivity to sarin compared to dry air. Differences in the properties of FPOL materials between [51,55] may result from the non-uniformity of the original FPOL (described in [55]).

A common characteristic of the carbon materials described above, developed in the early stages of research on acidic polymers, is their relatively high glass transition temperature, which translates into poor sensor response dynamics. The elevated T_g_ values arise from constraints in the rotation of the carbon chains, which are further increased by the incorporation of acidic functional substituents. Consequently, in subsequent years, there was a decline in interest in carbon materials in favor of polysiloxane and carbosilane chain-based polymers characterized by inherently lower T_g_ values.

### 2.2. Linear Silicon-Containing Polymers with Aliphatic Fluoroalcohol Substituents

New avenues for the development of acidic polymers were sought among polysiloxane chain polymers. Polysiloxanes are characterized by exceptionally low glass transition temperatures resulting from the significant freedom of rotation around the Si-O-Si bond (e.g., for polydimethylsiloxane—PDMS—T_g_ is approximately −125 °C [57]). Additionally, these substances exhibit high chemical and thermal resistance.

**Figure 3:**
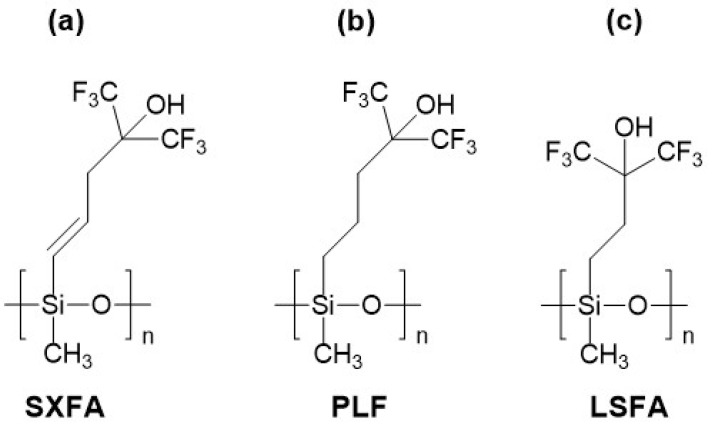
Structures of repeating units of some acidic polymers with fluoroalcoholic groups based on polysiloxane backbone: (**a**) SXFA, (**b**) PLF, and (**c**) LSFA.

The first described HBA polysiloxane material was poly(methyl [4-hydroxy-4,4, bis(trifluoromethy1)but-1-en-1-yl]siloxane dubbed SXFA, whose synthesis was described by Abraham et al. in 1995 [58]. The structure of SXFA is presented in Figure 3a. The material was obtained through the reaction of gaseous hexafluoroacetone with previously prepared polymethylallylsiloxane. In the SXFA structure, the HFIP group is responsible for creating acidic hydrogen bonds. In the work [58], the LSER coefficients of the SXFA material were also determined at a temperature of 25 °C, confirming its decidedly acidic nature. The SXFA material is characterized by a T_g_ at −44 °C [59,60].

The first reports on the use of SXFA in an acoustic wave sensor for the detection of organophosphorus compounds come from 1997–1998 [61,62]. In 1998, McGill et al. reported the application of SXFA in an SAW device with a resonant frequency of 250 MHz. An innovative polymer deposition method—matrix-assisted pulsed laser evaporation—MAPLE was used to achieve uniform layers of strictly controlled thickness and high stability. The study tested a sensor with an SXFA layer thickness causing a resonant frequency decrease of 250 kHz. Additionally, covalently attached monolayers of SXFA were deposited on quartz substrates of SAW transducers (functionalization caused a resonant frequency decrease of 10 kHz). Both prepared sensors were tested in the presence of DMMP vapors, obtaining a signal of 65 kHz for 10 mg/m^3^ for the first sensor and approximately 14 kHz for 348 mg/m^3^ for the sensor with the SXFA monolayer. This means that the sensor with a thicker polymer layer exhibited significantly higher sensitivity than the monolayer sensor. On the other hand, comparing the response dynamics of both sensors, the sensor with the SXFA monolayer demonstrated significantly better properties (the signal reached 80% of its value in about 1 s, whereas for the MAPLE-deposited layer sensor, it was approximately 30% in 10 s).

Furthermore, the results regarding the detection of DMMP using a sensor with SXFA layers produced by spray coating and MAPLE methods were also described in 2003 [63]. In this case, trace concentrations of DMMP were detected using sensors with two-port SAW resonators with a central frequency of 417 MHz. Despite applying layers of different thicknesses (for spray coating—15 nm/500 kHz; and for MAPLE—35 nm/1200 kHz), similar sensor sensitivities were achieved (DMMP at a concentration of 0.1 mg/m^3^ generated a signal of approximately 2.5 kHz stabilizing within a few seconds, but the details of the measurement conditions were not provided in the study). This was probably due to the influence of the applied deposition technique on the properties of SXFA.

Another important work regarding SXFA was published by Wang et al. in 2011 [59], which aimed to determine the optimal parameters for the operation of the SXFA sensor. In this study, the values of the shear modulus of the polymer were experimentally determined (approximately 9 GPa), classifying it as glassy–rubbery material. By determining the value of the shear modulus, it was possible to determine the optimal thickness of the polymer coating for an SAW device with a given resonant frequency. The criterion here was to maintain a relatively high sensor signal with acceptable attenuation (below 10 dB) in the DMMP concentration range from 0 to 1000 mg/m^3^. The theoretically determined values were later confirmed experimentally. The optimal thickness of the SXFA layer for a 300 MHz SAW delay line was 40 nm. The prepared sensor exhibited a signal value of 1600 Hz for a DMMP concentration of 0.6 mg/m^3^ (RH = 23%, temperature 14 °C). The calculated limit of detection—LOD—value for this compound was also provided in the study, equal to 0.004 mg/m^3^. Additionally, the study analyzed the relationship between the sensor signal and the response and regeneration times as a function of measurement temperature.

The use of the SXFA material was also described by Singh et al. in 2016 [64]. In this study, the results of testing a sensor based on a single-port SAW resonator with a center frequency of 433 MHz were presented. This resonator is available on the market as an element used in radio communication and is therefore mass-produced and widely available. A layer of SXFA was deposited on the resonator, causing a shift in the resonance frequency by approximately 200 kHz, obtaining a signal for DMMP of around 4.3 kHz at a concentration of 1 ppm at 30 °C. Using the sensor, DMMP and other organic vapors (methanol, benzene, diesel vapor) were detected at various temperatures (from −20 to 70 °C). Due to differences in sensor sensitivity at different temperatures (mainly resulting from changes in analyte solubility in the polymer), the detection cycle of each analyte composed of measurements at different temperatures yielded a specific signal pattern. Based on these data, using principal component analysis and artificial neural network algorithms, it was possible to identify individual analytes.

It is also worth noting the work published by Pan et al. in 2020 [60], which presented an in-depth analysis of the impact of temperature, humidity, other organic compounds, smoke, and aging effects of the SXFA layer on DMMP detection. In this study, a significant enhancement in DMMP sensitivity in humid gas conditions was emphasized. During measurements conducted at a temperature of 26 °C, a signal increase of over 13 times was observed for 1 mg/m^3^ DMMP with varying gas relative humidity from 30 to 80%. This is also the first study to report such a significant impact of humidity on SXFA sensors.

The SXFA material continues to garner significant interest, with new studies emerging continuously regarding its application in acoustic wave sensors for detecting organophosphorus compounds. An example is a study from 2024, which investigated the response of an SAW delay line coated with SXFA to GB and DMMP vapors [65].

In 2000, a new linear polysiloxane with HFIP groups, polymethyl [3-(1,1,1,3,3,3-hexafluoropropan-2-ol)propyl]siloxane (PLF), was introduced (Figure 3b) [66]. Structurally and in terms of properties, this polymer was similar to the previously synthesized SXFA material. LSER model coefficients were determined for PLF in the cited work and compared with other sensor polymers. PLF was first used in an acoustic wave sensor (Love-type SAW) for DMMP detection in 2001 by Zimmermann et al. [67]. Unlike Rayleigh wave sensors, Love wave sensors have a guiding layer where the acoustic wave, polarized parallel to the waveguide plane, is generated, and the sensor layer is coated on its surface. The PLF polymer used in the study had a grafting ratio of 73% and a T_g_ of −17 °C. The study presented DMMP detection results, using a sensor with a center frequency of 111 MHz and a 15 nm PLF layer. A signal of 2 kHz at a DMMP concentration of 0.35 ppm (measurements conducted in dry nitrogen at 25 °C) was obtained. Additionally, the study determined the diffusion coefficient of DMMP in PLF at 25 °C based on the sensor’s response dynamics.

In the works [68,69], the same scientific team described the use of Love-type sensors with PLF for GB and DMMP detection. The study tested a series of Love wave sensors with variations: in the thickness of the guiding layer made of amorphous SiO_2_ (4.6 and 6 μm), in center frequency (from 87 to 115 MHz), and in PLF sensor layer thicknesses (20 and 40 nm). Particularly valuable are the comparative detection results of GB and DMMP. These studies revealed significant differences in the sensitivity and response dynamics of sensors to vapors of these substances. In the case of DMMP, the sensors exhibited much higher sensitivity than for GB. The SAW sensor, operating at a center frequency of 109 MHz equipped with a 20 nm PLF layer resulting in a frequency decrease of approximately 108 kHz, exhibited a response of about 3.5 kHz for a DMMP concentration of 2 ppm. In contrast, the response for GB was only approximately 1.5 kHz. The measurements were conducted at 25 °C. Additionally, a significantly longer stabilization of the response (15–20 min for DMMP and about 5 min for GB) was observed. The cited study also conducted an interesting analysis of the transient states of sensor signals, which also showed significant differences between GB and DMMP. Furthermore, the study determined the diffusion coefficient of GB in PLF. Additionally, in the work [68], important results of GB detection in dry and humid air were presented, indicating a significant decrease in sensor sensitivity in humid air. A decrease of up to 80% compared to the signal in dry air at a gas relative humidity of 50% was also reported in conference materials [70]. Nonetheless, despite such a significant decrease in sensitivity, the sensor still allows for the detection of this analyte. The observed decrease in the sensitivity of PLF sensors contrasts with the behavior observed in sensors with an acidic polymer such as FPOL [55] or SXFA [60]. In their case, humidity, on the other hand, caused a significant increase or no change in sensitivity to organophosphorus compounds.

Research results on PLF can also be found in studies published in subsequent years [71,72,73]. In 2013, Wang et al. [71] reported the results of testing the PLF sensor, and they described a sensor coated with a structurally very similar polymer, acronym LSFA (Figure 3c). PLF and LSFA were deposited on the surface of two-port SAW resonators with a center frequency of 434 MHz. The polymer layer thicknesses were very similar, measuring 20.5 and 21 nm for PLF and LSFA, respectively, causing a frequency change of approximately 750 kHz. Measurements conducted at 15 °C in dry nitrogen showed differences in the sensitivity of PLF and LSFA sensors. In the case of the PLF sensor under these conditions, a signal of 3.5 kHz was obtained for DMMP at a concentration of 1 mg/m^3^, while for the LSFA sensor, it was as high as 10 kHz. Additionally, the method of synthesis used to obtain PLF and LSFA in this study is noteworthy. These polymers were obtained by modifying an existing polysiloxane chain (polymethylhydrosiloxane—PMHS) through a platinum-catalyzed hydrosilylation reaction. The synthesis did not involve polymerization but only the introduction of a functional substituent containing a vinyl group (and a functional fluoroalcohol group) into the polysiloxane chain. This method proved to be very useful and offered significant possibilities for obtaining polysiloxanes for sensor applications. In subsequent years, it gained importance in the research of new sensor materials. Such synthesis is relatively easy to carry out and allows for the one-step fabrication of functional polysiloxanes, not only with acidic properties. This approach has been replicated multiple times in the research of other sensor materials.

In 2015, Long et al. [72] analyzed the differences in the PLF sensor’s response to live CWAs (GB, HD) and their simulants (DMMP and 2-chloroethyl ethyl sulfide-CEES). They employed two-port SAW resonators with a center frequency of 434 MHz, onto which PLF was deposited in a layer causing a frequency decrease of approximately 800 kHz. Measurements were conducted at a temperature of 25 °C. A direct comparison of the sensor response to DMMP and GB revealed significant differences in sensitivity, detection limit, and especially in response dynamics. The sensor response to DMMP vapors at a concentration of 1 mg/m^3^ reached 80% after about 50 s (steady-state response was 3.1 kHz). In the case of GB, the signal increased much more slowly, reaching 1.6 kHz after 3 min for an analyte concentration of 1 mg/m^3^. The GB signal did not reach a steady state even after a 16 min exposure to a concentration of 10 mg/m^3^ of this gas.

### 2.3. Linear Silicon-Containing Polymers with Phenol and Fluorophenol Substituents

The analysis of the solvation properties of phenols and aliphatic alcohols leads to the conclusion that phenols inherently constitute much better hydrogen-bonding acids and weaker bases than the latter [74]. Introducing fluorine atoms into molecules causes an increase in acidity and a simultaneous decrease in basicity in both cases. Comparing the acidity of hexafluoroisopropanol (A = 0.77) with its aromatic counterpart (3,5-bis(trifluoromethyl)phenol, A = 0.82), it can be observed that the acidity of the latter is even higher [75].

In 1997, Grate et al. [76] described a hybrid material consisting of carbon segments (2,2-bis(3-propyl-4-hydroxyphenyl)hexafluoropropanes) with strong acidic properties linked by polysiloxane linkers, which provide plasticizing properties, lowering T_g_. This material is considered the first HBA polymer utilizing phenolic acid groups instead of the commonly used aliphatic fluoroalcohol groups. The motivation for using a carbon segment containing this bisphenolic group was likely a previous work [77], which considered a series of bisphenols and phenols (in their free form—not introduced into the polymer chain) as potential candidates for fabrication coatings for piezoelectric sensors. This study identified 2,2-bis(3-propyl-4-hydroxyphenyl)hexafluoropropanes as having outstanding acidity with low basicity.

**Figure 4:**
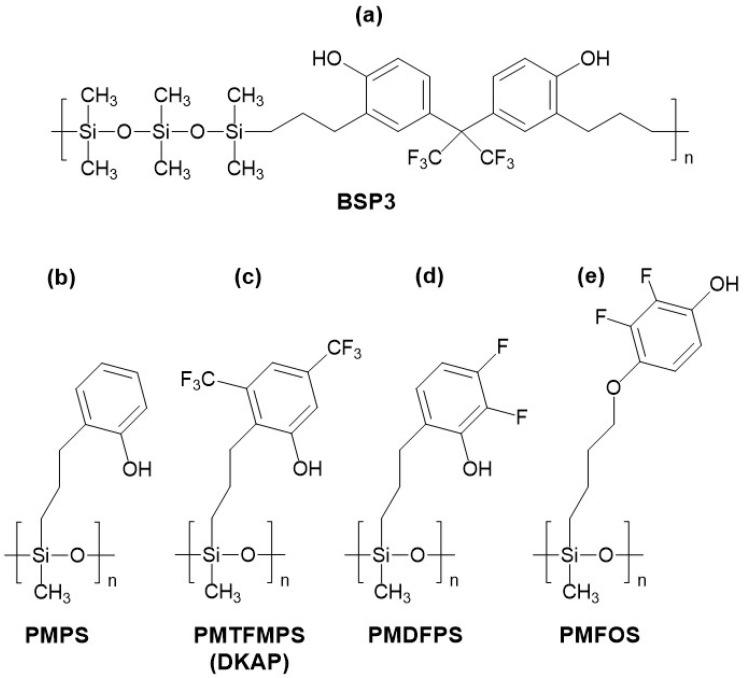
Structures of repeating units of some acidic polymers with phenolic or fluorophenolic groups based on polysiloxane backbone and acronyms used in research papers: (**a**) 2,2-bis(3-propyl-4-hydroxyphenyl)hexafluoropropanes) and polydimethylsiloxane hybrid material (BSP-3) (**b**,**c**) phenol (PMPS, PMTFMPS) and (**d**,**e**) fluorophenol modified polysiloxanes (PMDFPS, PMFOS).

The hybrid material described in [76] was synthesized in three variations differing in the amount of polysiloxane monomers in these linkers (the materials were labeled as BSP3, BSP6, and BSP80—the number in the name indicates the number of polysiloxane monomers in the linker, respectively 3, 6, and approximately 80, with BSP3 and BSP6 receiving the most attention in the study). The materials were synthesized through a platinum-catalyzed hydrosilylation reaction. The structure of the BSP3 material is depicted in Figure 4a. The cited study described the synthesis of new materials, detailed material studies, including determining their T_g_ temperatures (equal to 6 °C for BSP3 and −16 °C for BSP6), and preliminary studies of SAW sensors (DMMP vapor detection).

In a subsequent study published by the same scientific team [78], a more in-depth analysis of the suitability of new materials for use in SAW sensors was conducted. In this study, an experimental comparison of SAW sensors equipped with layers of four different HBA polymers, BSP3, BSP6, and previously described SXFA and FPOL, and polymers with other properties was conducted. Two-port SAW resonators with a center frequency of 200 MHz were used for measurements, onto which polymer layers causing a frequency decrease of approximately 250 kHz were deposited. Based on the research, it was concluded that the highest sensitivities to basic vapors were exhibited by sensors with SXFA and BSP3 materials (compared to sensors with nonpolar coatings such as PIB (poly(isobutylene)) and OV25 (polarizable phenyl groups), the sensitivity of SXFA and BSP3 sensors to basic vapors was about 10–20 times higher). At the same time, it was noted that the SXFA sensor was slightly more selective than BSP3. An analysis of sensor response times based on measurements conducted at 25 °C showed that SXFA, BSP3, and BSP6 sensors reached 90% of the steady-state response within 6 s. In contrast, the FPOL sensor was significantly slower (50% response from steady state within 6 s).

Despite nearly thirty years passing since the first synthesis of BSP3, research on this polymer continues. In 2015, Wang et al. [79] described an optimized sensor using BSP3. Sensor parameter optimization involved the use of an SAW device with an appropriate center frequency and the application of a sufficiently thick polymer layer (calculations indicated an optimal frequency lower than 500 MHz and a layer thickness less than 100 nm). Measurements utilized a two-port SAW resonator with a resonant frequency of 300 MHz, and a BSP3 layer thickness of 76 nm, which resulted in a resonant frequency reduction of approximately 170 kHz upon layer deposition. DMMP measurements were conducted at a temperature of 20 °C and relative humidity—RH—of 35%. Sensitivity to DMMP was obtained at 3.09 kHz/mg/m^3^ (in the DMMP concentration range of approximately 0–2 ppm), with a detection limit of 0.004 mg/m^3^ (calculated based on the sensor’s sensitivity at the beginning of the concentration curve and baseline noise amplitude). Similar to earlier reports, this study also observed a strongly nonlinear DMMP concentration characteristic. To date, the values of the inherent sensitivity and DMMP detection limit presented in the above study remain among the best achieved for a sensor with an absorptive sensor layer made of HBA polymers (without preconcentration).

Du and coworkers developed an interesting family of polysiloxanes with phenolic and fluorophenolic substituents [80,81,82]. This work described the synthesis and results of sensor studies with polymers with the acronyms PMPS (first synthesized as a reference in the research on HBA spatial architecture materials [83]), PMDFPS, and PMTFMPS (also referred to as DKAP, mentioned for the first time in 2006 [84]; however, the cited work did not include the synthesis or characterization of the material). These polymers were functionalized with substituents containing phenolic, bis(trifluoromethyl)phenolic and difluorophenolic groups, and, respectively (the structures of the materials are shown in Figure 4b–d). Among the polymers mentioned above, PMTFMPS exhibited the best sensor properties in terms of sensitivity, DMMP LOD (0.11 ppm at 20 °C), and resistance to interferents (acetone, dichloroethane, hexane, toluene, water). Additionally, this sensor demonstrated a good response dynamic (80% of the steady-state value was achieved within 60–80 s). Since this polymer was tested using a quartz microbalance (with a center frequency of 8 MHz), it is difficult to compare the sensitivity and DMMP detection limit to the results obtained for other polymers using SAW sensors. The PMTFMPS sensor also showed a decrease in sensitivity to DMMP in humid gas (RH = 77%) of about 40% compared to the signal in dry air.

In 2014, the results of SAW sensor studies coated with PMTFMPS (referred to as DKAP in the original study) were presented [85]. The sensor was used for the detection of DMMP, GB, and GD. In this study, a dual-port SAW resonator with a center frequency of 434 MHz and a polymer layer causing a frequency drop of 1.2 MHz was used. Similar to the PLF sensor reported in [72], significant differences in response dynamics between live agents and their simulants were obtained. The DMMP signal stabilized depending on the concentration from 40 to 150 s, while in the case of GB and GD, it took several minutes. Nevertheless, the transient response values measured after 3 min from the onset of exposure to these substances were substantial (3.3 kHz for GB and 4.3 kHz for GD, respectively), enabling unequivocal qualitative analysis. Differences in the sensor’s response dynamics to different substances were attributed to the slower diffusion rate of live CWA vapors due to structural differences (the molecules of sarin and soman are much larger than DMMP and require more energy to create cavities between polymer chains). The measurement temperature (20 °C), likely close to the glass transition temperature of this material (T_g_ = 20 °C [86]), probably also influenced the dynamic characteristics of the PMTFMPS sensor.

Subsequent studies regarding the application of polysiloxanes with fluorophenolic substituents were published in the years 2021–2022 [73,87,88]. These works described the synthesis, characterization of sorption properties, and application in SAW sensors of the [4-(2, 3-difluoro-4-hydroxyphenoxy) butyl] siloxane—PMFOS. PMFOS is structurally very similar to PMDFPS, but it has a hydroxyl group with an acidic hydrogen atom in a more exposed para position relative to the alkoxy linker with the polysiloxane chain, unlike its analog. The structure of PMFOS is presented in Figure 4e. In the study [73], a sensor with PMFOS was described, based on a dual-port SAW resonator with a center frequency of 195 MHz, and its parameters were compared with an identical resonator coated with PLF (also synthesized within this study, both materials were characterized by a similar grafting ratio of approximately 30%). The responses of sensors with polymer layers causing a frequency drop of 100 kHz were analyzed. Both sensors exhibited similar sensitivity (signal on DMMP at a concentration of 5 ppm at a temperature of 30 °C, respectively 4.5 and 4.2 kHz for PMFOS and PLF) and detection limits for DMMP (in both cases, approximately 13 ppb) and triethyl phosphate (TEP—another nerve CWA simulant). The analysis of interference effects indicated that the sensors enable the selective detection of organophosphorus compounds, although the PMFOS sensor signal may be disrupted by triethylamine vapors. Nonetheless, this sensor also showed a reduced impact of water vapor on sensitivity to DMMP compared to the PLF sensor.

### 2.4. Silicon-Based Materials with Hyper-Branching and Spatial Architecture

**Figure 5:**
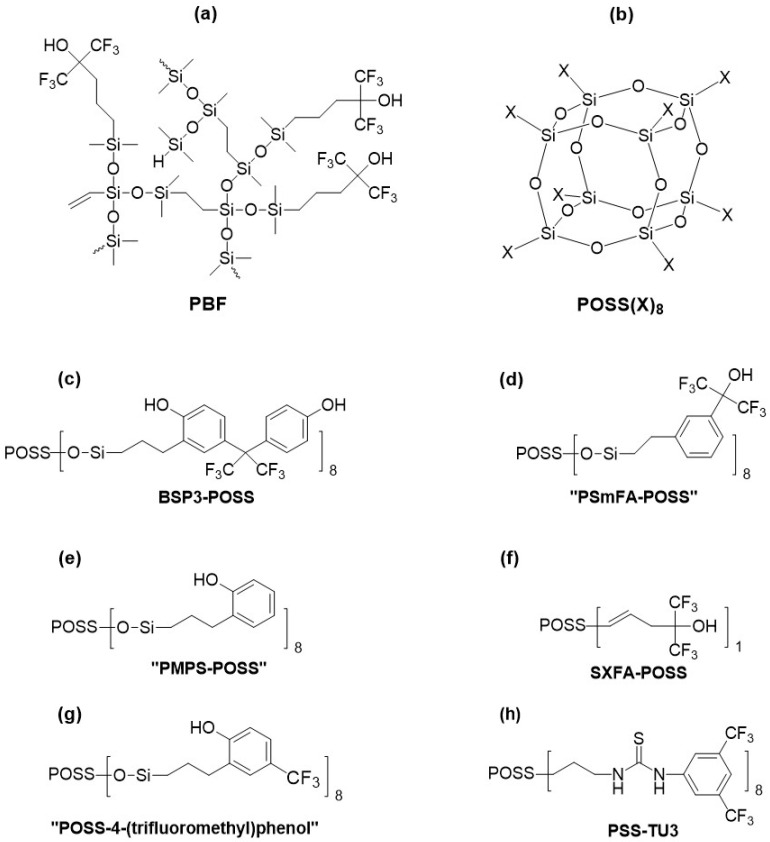
Structures of some acidic materials with branched or spatial architecture and their acronyms (acronyms in quotation marks were assigned by authors of this review due to perceived similarity to substituents of spatial materials and some compounds with linear architecture or lack of acronyms in original works): (**a**) branched polysiloxane with HFIP substituents (PBF); (**b**) polyhedral oligosilsesquioxane core (POSS) used in the synthesis of spatial analogs of: (**c**) BSP3 (BSP3-POSS), (**d**) PSmFA (PSmFA-POSS), (**e**) PMPS (PMPS-POSS), (**f**) SXFA (SXFA-POSS as well as materials containing (**g**) 4-(trifluoromethyl)phenol and (**h**) thiourea based substituent (POSS-4-(trifluoromethyl)phenol, PSS-TU3).

In parallel with the initial studies on PLF in 2000, reports emerged on a hyper-branched version of this material [66], describing a polymer referred to as PBF (its structure is presented in Figure 5a). The motivation behind utilizing polymers with dendritic or hyper-branched structures stemmed from the potential for a higher density of functional groups, which could lead to greater sensor sensitivity while simultaneously allowing for a lower density of segmental entanglements, thereby facilitating analyte diffusion within the layer volume. However, the cited work did not present the synthesis or application of PBF in acoustic wave sensors but solely characterized its solvating properties aimed at detecting organophosphorus compounds.

A series of significant works on branched acidic polysiloxane was published by the Hartmann-Thompson group from 2004 to 2008 [83,89,90]. In the 2004 study [89], several acidic sensing materials were described based on two cross-linked polysiloxane cores: one with allyl groups (labeled as HB-PCS) and the other with dimethylhydrosilane groups (labeled as HB-PCSOX). These cores were synthesized via platinum-catalyzed hydrosilylation reactions of macromolecules with respective molecular weights of Mw = 6322 g/mol (polydispersity index of 1.97) and Mw = 2913 g/mol (polydispersity index of 2.16). Subsequently, the macromolecules were functionalized with phenolic and fluoroalcohol groups, resulting in materials analogous to SXFA, FPOL, BSP3…80, and PMPS (in the original work, the latter material was named as “functionalized with non-fluorinated allylphenol”). These polymers were applied to SAW sensors with a center frequency of 500 MHz (the polymer layer caused a frequency drop of 500 kHz). The sensors’ responses to DMMP at a concentration of 0.5 mg/m^3^ and a temperature of 28 °C were tested. The highest response values were obtained for the BSP3 analog (3629 Hz) and the PMPS analog (2740 Hz). The response values for the SXFA and FPOL analogs were 638 and 530 Hz, respectively. The study also analyzed the stability of the sensor properties over time, revealing a significant decrease in DMMP sensitivity for most sensors over approximately six months (a 70–80% loss of sensitivity). Only the sensor with the FPOL analog showed no significant sensitivity changes. The topic of branched materials based on a similar core structure, as described in the cited study, was also presented in 2008 [90]. This publication introduced materials functionalized with polyester substituents with moderate acidic properties.

In 2007, materials utilizing polyhedral oligosilsesquioxane cores (labeled as POSS, structure is presented in Figure 5b) were introduced [83]. The POSS’ functionalization involved introducing functional substituents known from linear acidic polymers such as BSP3, PSmFA, PMPS, and SXFA (Figure 5c–f); the second and third materials were not named as analogs of PSmFA and PMPS in the original work, but such a comparison was decided for the sake of greater transparency of this review). Since the functionalized POSS were in the form of solids or rubbers at room temperature, they were dissolved in an inert carrier polymer (poly(methylphenyl)carbosilane) to enable the formation of an absorption layer on the sensor surface. Consequently, compositions with a specified percentage content of functionalized POSS in the polymeric filler were applied to the sensors. The materials were tested using SAW sensors with a center frequency of 500 MHz, yielding similar results to the HB-PCS and HB-PCSOX analogs described above [89] although significantly inferior to classical linear counterparts, which were also synthesized and examined as part of this study. Nevertheless, the long-term stability of the layers significantly improved, enabling the preservation of 40–65% sensitivity over a 6-month testing period. 

Materials based on POSS continue to attract significant interest to this day [91,92]. In 2020, Kim et al. [91] described a material functionalized with substituents containing 4-(trifluoromethyl)phenol groups at the corners of the POSS cage (the structure of this material is presented in Figure 5g as POSS-4-(trifluoromethyl)phenol). The material was tested on SAW devices with a center frequency of 250 MHz using GB, GA, and DMMP. The following response values were obtained: for GA at a concentration of 50 mg/m^3^, approximately 11 kHz; for GB at a concentration of 55 mg/m^3^, approximately 2 kHz; for DMMP at a concentration of 63 mg/m^3^, approximately 4.1 kHz. The stabilization times of the responses for all analytes were similar, ranging from 40 to 50 s. However, the study did not specify the thickness of the deposited layers or the temperature at which the measurements were conducted. Additionally, the stability of the sensors over time was checked, revealing that the sensors maintained an efficiency ranging from approximately 80% to 65% over 180 days.

In 2023, Bae et al. [92] described the synthesis and investigation of thiourea-decorated POSS with fluorinated substituents that increase acidity, named PSS-TU3 (its structure is presented in Figure 5h). Unlike most previously used materials, in this case, there were no hydroxyl groups, but hydrogen atoms bonded to the nitrogen atoms in secondary amines of the thiourea substituent. The SAW sensor with a center frequency of 250 MHz provided a signal of 14.725 kHz per 1 ppm DMMP. However, this study also lacks crucial data regarding the measurement conditions, which would enable comparison with other works.

### 2.5. A Summary of the Review of HBA Polymers

The signal value of the acoustic wave sensor with an absorptive sensor layer is influenced by many factors. The various sensitivity values, detection limits, and dynamic parameter reported in experimental studies do not solely result from the different solvating properties of individual polymers but also from differences in the acoustic wave devices used and measurement conditions. The most important parameters affecting the sensor signal include the following: the type of acoustic wave device, center frequency (sensitivity increases with the square of the frequency), sensor layer thickness, analyte concentration range (due to the nonlinear characteristics of HBA sensors in the sorption of basic vapors, sensitivity changes with concentration), measurement temperature, and humidity of the analyzed gas. The variations in these parameters across different scientific studies pose challenges for directly comparing the sensitivity values, detection limits, or dynamic properties of individual sensors between publications.

Nevertheless, to provide an overview of the progress in research on acoustic wave sensors with acidic polymers, the basic parameters of some sensors reported in the literature are gathered in Table 2. Table 2 presents signal values for particular analyte concentrations, sensitivities, detection limits for organophosphorus analytes, and information characterizing the acoustic wave devices and measurement conditions. The comparison is restricted to displaying results obtained for surface acoustic wave devices, as they are prevalent in the literature. All analyte concentrations have been converted from the original units used in the reports to mg/m^3^ to enhance clarity in comparison.

## 3. Discussion and Conclusions

Research on acoustic wave sensors equipped with absorption sensor layers for nerve CWAs’ detection has been going on for about 40 years. During this time, many materials were developed that differed in the composition of the polymer chain used, their spatial architecture, and the type of functional substituents. Despite this, the general idea behind the inventors of these materials, closely related to the selective mechanism of sorption of organophosphorus compounds, has remained unchanged. The researchers’ intention was and still is for each newly synthesized material to be characterized by high hydrogen bond acidity with low basicity, a high diffusion coefficient of analyte vapors in the polymer volume (achieved by low T_g_), and stability over time. The latest achievements show that researchers are achieving considerable success in this field, and the construction of sensors detecting nerve CWAs at concentrations of single ppb is within the reach of the discussed analytical technique.

Two issues that appear in scientific works in this field require a longer comment: the influence of moisture on the sensitivity towards organophosphorus compounds and the long-term stability of polymer layers. 

There are few works that present the results of systematic studies of the influence of moisture. As some of them show, the co-existing gas humidity is very important and can lead to both a significant increase in the sensitivity towards nerve CWAs and its decrease. Taking into account that significant amounts of water vapor are found in every real sample, the analysis of the influence of humidity should be a mandatory and more exposed experimental stage in every work in this field. By analyzing the available materials, it can be concluded that the influence of moisture depends on the properties of specific materials. In works describing FPOL, BSP3, and SXFA sensors, an increase in sensitivity or no effect on the signal was observed [51,55,60]. However, in the case of PLF, PMPS, PMDFPS, and DKAP, a significant decrease in sensitivity to DMMP was observed (up to 80% at gas humidity RH = 50% [68,73,80,82]). What is more surprising is that extremely different behaviors are observed for materials that are very similar in structure, e.g. SXFA and PLF [60,68]. 

The stability of polymer layers over time is another important problem that is raised in very few works. In some of them, the produced layers are stable and the sensors retain good properties for several months [55,60]. In others, a drastic decrease in sensor sensitivity is observed within even a few days [83,89,91]. Moreover, it is interesting that these extremely different results often concern the same materials but applied using different methods and tested by different scientific teams. The reasons for these differences cannot be attributed to the inherent properties of these polymers. The differences in the stability of the layers probably result from the way they are applied and attached to the substrates. Features specific to macromolecules, such as the distribution of molar masses, the degree of cross-linking, or the grafting ratio, may also be important. These parameters are not reported in all works in the field, but they may have a significant impact on the stability of polymer layers (e.g., through significant material volatility in the case of short chains and poor cross-linking).

Despite many years of intensive development and research, it can be said that the acoustic wave sensor technique in the field of nerve CWAs’ detection has not gained as much popularity as, for example, ion mobility spectrometry (IMS) or flame photometry (FP). However, in recent years, several commercial devices utilizing acoustic wave sensors have been developed [93], and some of them are still available [94]. In the target application so far, which is portable gas analyzers used by a small number of specialized soldiers/first responders, the main advantages of acoustic wave sensor technology did not play such an important role. Perhaps that is why they were not much of a commercial success.

Analyzing the current requirements and trends related to field CWAs’ detection, one can notice a potential opportunity for the wider use and further development of acoustic wave sensor technology. Recently, new military fields of application have appeared, such as wearable detectors and distributed sensor networks. In this case, acoustic wave sensors seem to be promising candidates. In these applications, the following functional features are very important: low operational requirements, autonomous operation, low unit cost, and high miniaturization potential. These requirements can be met very well by acoustic wave sensor technology.

An additional opportunity lies in the development that has occurred in the field of acoustic wave transducers in recent years. This is especially true for high-frequency devices such as the FBAR [95,96], the use of which may further improve the detection of target analytes (since the sensitivity of this type of sensor increases with the square of the operating frequency) [29].

## Figures and Tables

**Table 1:** LSER descriptor for nerve CWAs and their simulant—DMMP. CWAs’ descriptors were determined through QSPR calculations [34]. DMMP descriptors are taken from [35].

Substance Codename/CAS	LSER Descriptors					Source
	E	S	A	B	L	
GA/77-81-6	0.34	1.14	0.05	1.29	4.78	[34]
GB/107-44-8	0	0.74	0	0.81	3.10	
GD/96-64-0	0	0.74	0	0.81	4.35	
VX/50782-69-9	0.50	1.19	0	1.63	7.53	
DFP/55-91-4	−0.05	0.42	0	0.86	3.83	
DMMP/756-79-6	0.21	1.62	0	1.01	3.90	[34]

**Table 2:** Comparison of basic parameters of some acoustic wave sensors encompassing both organic and silicon-containing materials.

Group of Materials	Name (Acronym)	Type of SAW Device/Center Frequency [MHz]	Layer Thickness [nm]/[kHz]	Temp. [°C]/Humid. [%]	DMMP Conc. [mg/m^3^]	Response [kHz]/ LOD [mg/m^3^]	Source
Aliphatic fluoroalcohol substituents, linear backbone	FPOL	Delay line/158	N/A */250	30/0 **	1.1	1.3/1	[55]
	SXFA	Delay line/150	N/A/N/A	13/47	0.8	1.6/0.12	[60]
	SXFA	Resonator/417	15/500	N/A/N/A	0.1	2.2/N/A	[63]
	SXFA	Delay line/300	38/N/A	14/23	0.6	1.6/0.004	[59]
	PLF	Resonator/434	21/765	15/0	1	3.2/N/A	[71]
	PLF	Resonator/195	N/A/100	30/0	1.5	0.62/0.07	[73]
	LSFA	Resonator/434	20.5/740	15/0	1	10/N/A	[71]
Phenol and fluorophenol substituents, linear backbone	BSP-3	Resonator/300	76/170	20/35	1.5	4/0.004	[79]
	PMPS	Resonator/434	N/A/600	25/70	316	16/N/A	[80]
	DKAP	Resonator/434	N/A/1200	20/N/A	1	11/N/A	[85]
	PMFOS	Resonator/195	N/A/100	30/0	1.5	0.5/0.07	[73]
Hyper-branched or spatial architecture	HB-PCSOX-BSP3	N/A/500	N/A/500	28/N/A	0.09	3.6/N/A	[89]
	HB-PCSOX-PMPS	N/A/500	N/A/500	28/N/A	0.09	2.7/N/A	[89]
	PSS-TU3	Delay line/250	N/A/N/A	N/A/N/A	1.0	14.7/N/A	[92]

* Data not available. ** An RH of 0 indicates that the original work stated that the measurements were performed in “dry” gas.

## Data Availability

The data presented in this study are available on request from the corresponding author.
